# Electronically Rotated Electrical Capacitance Tomography Sensing and Image Reconstruction Based on Transformer Model

**DOI:** 10.3390/s26206385

**Published:** 2026-10-09

**Authors:** Chengcheng Lu, Ze Yao, Lu Ma, Hongli Hu

**Affiliations:** 1 Aviation Technology Key Laboratory for Full-Scale Aircraft Structures Static/Fatigue, Aircraft Strength Research Institute of China, Xi’an 710065, China; luchch0114@stu.xjtu.edu.cn; 2 School of Electrical Engineering, Xi’an Jiaotong University, Xi’an 710049, China; yaoze@stu.xjtu.edu.cn (Z.Y.); ma.lu@xjtu.edu.cn (L.M.)

**Keywords:** electrical capacitance tomography, image reconstruction, transformer model

## Abstract

**Highlights:**

**What are the main findings?**

- Proposes the ER-ECT technique, which uses electrode subdivision and combined excitation strategies to increase the number of independent measurements.
- Applies the Transformer model with self-attention mechanism to directly solve the nonlinear mapping problem in image reconstruction.

**What are the implications of the main findings?**

- Improves the image quality and accuracy compared to traditional methods.
- Enhances the robustness against noise, as verified by both simulations and experiments.

**Abstract:**

Electrical capacitance tomography (ECT) is a noninvasive imaging technology based on capacitance sensors mainly used for measuring and imaging nonconductive fluid in multiphase flow. However, ECT has underdetermined and nonlinear problems due to limited sensor electrodes, which pose challenges for image reconstruction. Therefore, this paper uses the sensing technology based on electronically rotated electrical capacitance tomography (ER-ECT) to increase the number of independent measurements by combined excitation to alleviate the underdetermined problem. At the same time, ER-ECT combined with the Transformer model is used to reconstruct the dielectric constant distribution image from sensor measurement data, and the self-attention mechanism is used to solve the nonlinear mapping problem of ECT. The experimental results show that the proposed method is superior to the traditional method in terms of relative error and correlation coefficient and improves the quality and accuracy of image reconstruction of the sensor system.

## 1. Introduction

Electrical capacitance tomography (ECT) is an important branch of electrical imaging technology. ECT has received extensive attention and in-depth research in pharmaceutical production, oil and gas transportation [1], and environmental monitoring because of its instantaneous sensing response speed, high electrical characteristic contrast [2], low safety risk, and noninvasive measurement characteristics [3]. In the oil and gas industry, ECT shows its application potential in regulating transportation and separation processes by measuring and imaging pipeline multiphase flows [4].

ECT technology has a 30-year history [5]. The core principle of ECT is to measure the capacitance between the electrodes, reverse the dielectric constant difference in the measured area, reconstruct the media distribution, and obtain the volume fraction of each phase. The electric field distribution of ECT is susceptible to the significant influence of conductive and dielectric materials in the measurement area, resulting in a complex nonlinear relationship between the capacitance signal and the volume fraction of each phase in the area to be measured. This kind of nonlinear mapping often causes artifacts and boundary blur in the reconstructed image, which limits the imaging quality. In addition, ECT image reconstruction also faces a serious underdetermined problem: the amount of capacitance signal data is far less than the number of pixels required for reconstructing the image, resulting in the inability to obtain a unique solution when the mathematical model is solved, and only the approximate reconstruction will be achieved through the optimal solution.

Although ECT technology has shown broad application prospects, the underdetermined and nonlinear problems of the image reconstruction process have not been effectively solved. Traditional reconstruction algorithms, such as linear back projection (LBP) [6], Landweber iteration (LIA) [7], and Tikhonov regularization (TRM) [8], perform poorly in dealing with complex media distribution and noise interference, and the quality of reconstructed images is limited. Researchers have tried to address these challenges in recent years through hardware and algorithm improvements. In terms of hardware, optimizing the number of electrodes increases independent measurements. However, there is a contradiction between the number of electrodes and the measured signal-to-noise ratio [9]. Without increasing the physical electrode, alleviating the underdetermined problem has become a new research direction. Researchers use the mechanical rotation of ECT sensors to make 16 electrodes reach the level of 32 electrodes, thus avoiding the problem of signal-to-noise ratio decline caused by the increase in the number of sensors [10,11]. However, there are problems such as complex physical structure and time-consuming measurement. On this basis, the effect of mechanical rotation will be realized by series switching between electrodes, simplifying the mechanical structure and realizing fast switching [12].

In terms of image reconstruction algorithms, machine learning and neural networks have provided a new perspective for ECT image reconstruction in recent years [13]. Researchers use machine learning and neural network algorithms to establish a nonlinear mapping between capacitance value and medium distribution [14,15]. These methods do not depend on sensitivity theory and directly establish the relationship between object field distribution and reconstructed image but often require a large number of training samples and prediction samples [16,17,18].

Aiming at the above problems of ECT technology, this paper proposes a novel electrical rotated electrical capacitance tomography (ER-ECT) technology. This work combines the ER-ECT measurement framework with a Transformer-based deep learning model for image reconstruction. ER-ECT dramatically increases the number of independent measurements of ECT and alleviates the under-determinedness problem by subdividing the electrode and implementing the combined excitation strategy. ER-ECT-Transformer (ER-ECT-T) uses the self-attention mechanism of the Transformer model to directly learn the nonlinear mapping relationship between capacitance value and dielectric constant distribution, breaks through the limitations of traditional algorithms in nonlinear modeling, and realizes image reconstruction under small samples through optimization strategies. In this paper, the performance of ER-ECT and ER-ECT-T is verified by simulation and static experiments and compared with traditional methods. The results show that the proposed method improves image reconstruction quality and anti-noise performance.

## 2. Methodology

### 2.1. ECT Principle

The purpose of the ECT measurement system is to accurately obtain the capacitance value between the electrode plates, and the inversion of the capacitance value obtains the distribution of the dielectric material in the area to be measured. In an ECT measurement system with *N* electrodes, the electrode plates are arranged around the area to be measured. Theoretically, the capacitance between all electrodes can be measured. The number of independent measurements is:

(1) $$\left(\begin{array}{c} N\\ 2 \end{array}\right)=\left(N\times \left(N-1\right)\right)/2$$

The capacitance *C_ij_* is usually the charge ratio between electrode *i* and electrode *j* to the excitation voltage. For example, the electrode *i* is excited by the voltage *V_i_*, and the rest remain grounded. When the voltage is 0, the capacitance *C_ij_* can be calculated:

(2) $$C_{ij}=Q_{j}/V_{i}$$

Among them, *Q_j_* is the total amount of charge induced on electrode *j*, and *V_i_* is the excitation voltage of electrode *i*. The excitation voltage is known, and the calculation of the charge *Q_j_* needs to introduce the correspondence between the electric field and the dielectric constant. According to the Gauss theorem, the charge on the electrode *j* can be calculated by the flux of the electric field *E* on the electrode surface *S_j_*:

(3) $$Q_{j}=\oint_{s_{j}}\varepsilon \left(x,y\right)E\left(x,y\right)dS$$

In the formula, $\varepsilon \left(x,y\right)$ is the dielectric constant distribution function, which reflects the dielectric properties of the material in the region to be measured. *E*(*x*, *y*) is the electric field distribution, which is determined by the excitation voltage and the dielectric constant distribution. *dS* is the area differential on the electrode surface *S_j_*.

The gradient of the electric field *E* through the potential *ϕ* is expressed as E=−∇ϕ and satisfies the Poisson equation:

(4) $$\nabla \cdot \left(\varepsilon \left(x,y\right)\nabla \phi \right)=0$$

In ECT’s practical work, the goal is to reverse the dielectric constant from the capacitance measurement value. This process is nonlinear. At present, the commonly used method is to correspond the capacitance measurement value with the dielectric constant change through the sensitivity matrix and establish the mapping relationship.

Sensitivity is defined as the partial derivative of the capacitance *C_ij_* concerning the permittivity distribution function $\varepsilon \left(x,y\right)$:

(5) $$S_{ij}\left(x,y\right)=\partial C_{ij}/\partial \varepsilon \left(x,y\right)$$

denotes the change rate of capacitance *C_ij_* when the dielectric constant at (*x*, *y*) in the area to be measured changes.

The area to be measured is usually discretized to form a grid cell, so the *k*th element of the sensitivity matrix *S* can be expressed as:

(6) $$S_{ij}\left(k\right)=\left(C_{ij}-C_{ij}^{\mathrm{ref}}\right)/\left(\varepsilon \left(k\right)-\varepsilon_{\mathrm{ref}}\left(k\right)\right)$$

In the formula, $S_{\mathrm{ij}}\left(k\right)$ denotes the sensitivity of the kth grid cell, $C_{\mathrm{ij}}^{\mathrm{ref}}$ is a uniform distribution when filled with a medium with a reference dielectric constant, the dielectric constant of the kth grid cell of $\varepsilon \left(k\right)$, and $\varepsilon_{\mathrm{ref}}\left(k\right)$ is the initial dielectric constant.

The corresponding relationship between the capacitance changes and the dielectric constant change is obtained, that is, the approximate correspondence described by the ECT inverse problem:

(7) C≈Sg

The goal is to solve the dielectric constant distribution *g*. However, since the number of independent measurements is generally much smaller than the number of grid elements to be represented, *S* is usually an irreversible matrix, so it cannot be solved directly. An appropriate solution algorithm is needed to make the approximate solution approach the real dielectric constant change. This is the underdetermined problem of ECT, which can be quantified as the condition number of the sensitivity matrix *S*:

(8) $$\vartheta \left(S\right)=S_{\sigma max}/S_{\sigma min}$$

Among them, $S_{\sigma max}$ and $S_{\sigma min}$ are the sensitivity matrix *S*’s maximum and minimum nonzero singular values, respectively. Due to the limitation of the number and position of the traditional ECT electrodes, the electric field coverage is effective, and $S_{\sigma min}$ is generally small, resulting in many conditions. The small disturbance of the capacitance measurement value will lead to the deviation of the solution of the dielectric constant change. Therefore, increasing the independent measurement quantity can alleviate the underdetermined problem of ECT and improve the overall measurement effect of ECT.

### 2.2. Electrical Rotated Electrical Capacitance Tomography

In the traditional 12-electrode ECT system, 12 independent electrodes are arranged to measure the area. According to (1), the independent measurement value is 66.

The ER-ECT technology proposed in this paper is improved based on the traditional 12-electrode ECT technology. The electrode rotation and dynamic grouping strategy are introduced to further subdivide the single electrode into three parts, forming a uniform distribution of 36 secondary electrodes. The independent measurement value is improved through the combined excitation strategy, and the condition number of the sensitivity matrix S is improved.

**Figure 1:**
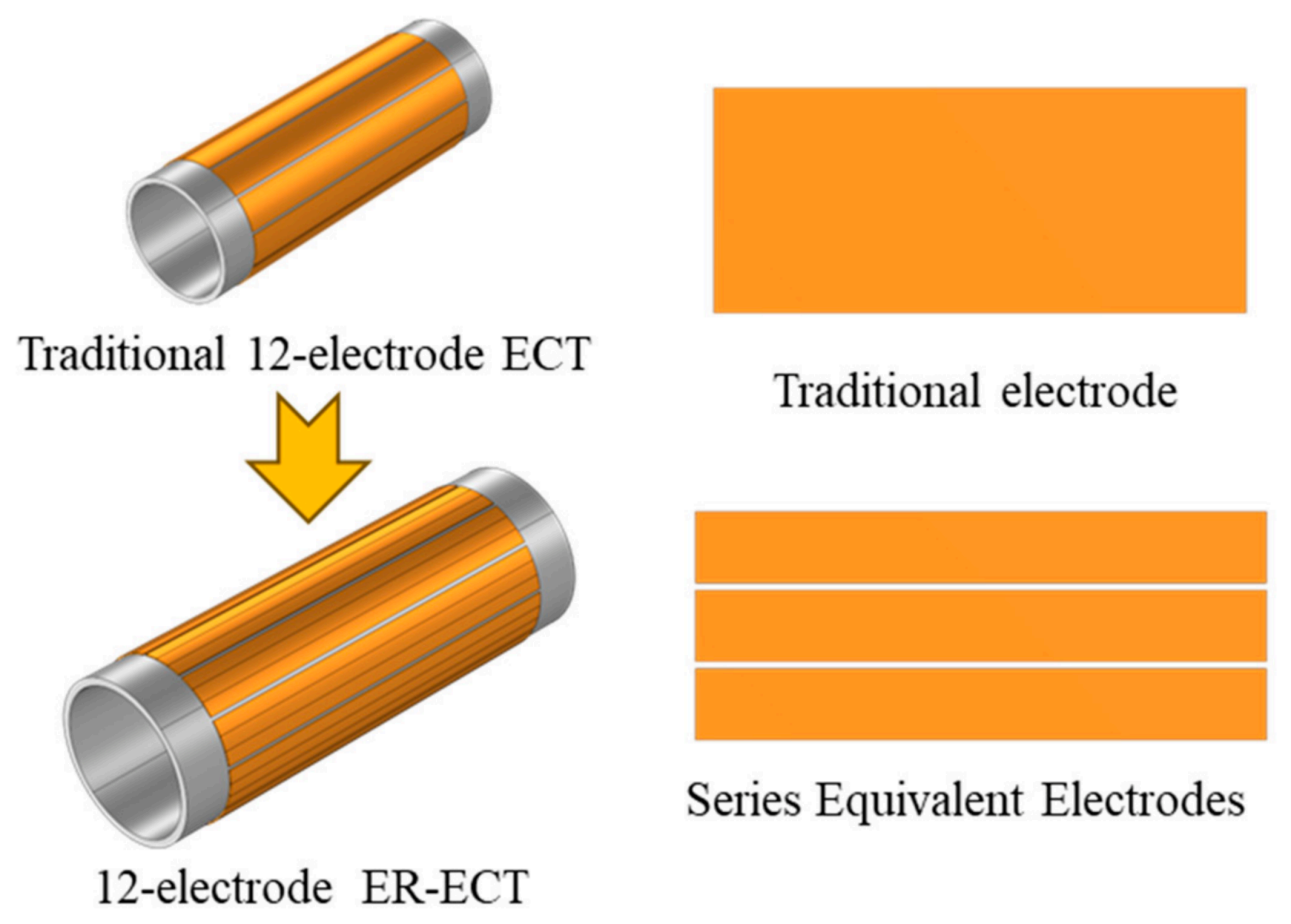
The schematic diagram of the structure of the ER-ECT sensor.

Figure 1 shows the specific measures of the improved method and the final sensor diagram and proposes a two-stage electrode combination excitation strategy based on the electrical rotating ECT sensor:

(a) Each electrode plate of the original 12-electrode ECT is marked as *Z_i_*, and the range of *i* is 1~12;
(b) the three subdivision electrodes subdivided by each *Z_i_* electrode plate are recorded as *Z_i__,j_*, and the value range of *j* is 1~3;
(c) each electrode plate of ER-ECT is denoted as *K_m,n_*. m represents the number of rotations, ranging from 0 to 2, and n is the serial number of the electrode plate, ranging from 1 to 12.

The strategy of the rule is expressed as:

(9) $$\left\{\begin{array}{c} K_{0,n}=Z_{n,1}+Z_{n,2}+Z_{n,3}\\ K_{1,n}=Z_{n,2}+Z_{n,3}+Z_{n+1,1}\\ K_{2,n}=Z_{n,3}+Z_{n+1,1}+Z_{n+1,2} \end{array}\right.$$

Three subdivided electrodes that make up the new *K_m,n_* electrode plate are connected in series each time. The above process can be regarded as the traditional 12-electrode ECT rotating twice, which realizes the spatial rotation of the electrode. Because of the change in the spatial position of the electrode, the measured values of the 12 electrodes are independent of each other after each rotation. On the right side, a new matrix *S_ER-ECT_* relative to the traditional 12-electrode sensitivity matrix is obtained:

(10) $$S_{ER-ECT}=\left[\begin{array}{c} S_{r1}\\ S_{r2}\\ S_{r3} \end{array}\right]$$

Among them, *S_r_*_1_, *S_r_*_2_, and *S_r_*_3_ are the traditional 12-electrode ECT sensitivity matrix with different angle configurations. After the ER-ECT rotates twice, the number of independent measurements is 198. Compared with the traditional 12-electrode ECT, the amount of independent measurement data increases by two times; that is, the number of rows of the sensitivity matrix under ER-ECT increases from 66 to 198, which provides more linear constraints on the change of dielectric constant, that is, the rank of the sensitivity matrix is improved, thus reducing the condition number of the sensitivity matrix and improving the underdetermination of the inverse problem. In theory, ER-ECT can bring higher-quality measurement results.

Compared with existing rotational ECT methods, the proposed ER-ECT has significant advantages:

(a) Mechanically rotating ECT: As the earliest rotating ECT scheme, this method rotates the entire sensor or electrode array via additional mechanical drivers such as gear sets or stepper motors to obtain measurements at different angles [19]. For instance, the rotatable 12-electrode ECT sensor proposed by Zhang et al. [20] drives the sensor to rotate in 6° steps via a stepper motor, but it suffers from complex hardware structure and slower measurement speed than conventional ECT. Meanwhile, mechanical motion inevitably introduces about 0.5% position error and vibration noise, degrading measurement accuracy. In contrast, our ER-ECT realizes virtual rotation via switching of subdivided electrodes, requiring no mechanical movement. It achieves faster measurement speed than mechanical rotating methods, with less mechanical error.
(b) Rotational Planar ECT (RPECT) [21]: It is designed for planar array ECT sensors, improving imaging quality by rotating the planar electrode array to increase measurement quantity. However, it is only applicable to planar array sensors and incompatible with widely used industrial pipeline circular ECT systems. ER-ECT, by contrast, is improved based on the traditional circular ECT sensor, compatible with existing pipeline ECT hardware and directly applicable to industrial pipeline multiphase flow measurement.
(c) Virtual rotation data augmentation method: A common data processing strategy in deep learning-based ECT reconstruction, it only performs rotation transformation on existing measurement data during processing to expand the training dataset and enhance model rotation invariance. However, it does not increase the number of independent measurements, essentially relying on the 66 measurement sets of traditional ECT and failing to alleviate the underdetermined problem at the hardware level. ER-ECT increases independent measurements from 66 to 198 via dynamic grouping of subdivided electrodes, adding effective hardware-level constraints and mitigating the underdetermined problem.

### 2.3. Transformer Model

ER-ECT improves the independent measurement value through the strategy of electrode rotation and dynamic grouping, which theoretically alleviates the underdetermined problem of ECT. However, there is still a big gap between the independent measurement value and the number of grids in the area to be measured. The capacitance measurement value and the dielectric constant in (7) are still nonlinear, and this nonlinear mapping is still one of the bottlenecks limiting the measurement effect. The Transformer model is one of the important models for dealing with complex dependencies in natural language processing and computer vision, and the effect is excellent. Therefore, this paper introduces the Transformer model and proposes a new method to break through the original linear assumption and establish the nonlinear mapping relationship between the capacitance measurement value and the dielectric constant obtained by ER-ECT to realize the high-precision reconstruction of the dielectric constant distribution in the area to be measured, namely ER-ECT-T.

The Transformer model establishes the global dependence between the input data through the self-attention mechanism rather than relying on the sensitivity matrix. In this paper, the 198 capacitance-independent values of ER-ECT are used as the input sequence, and the dielectric constant distribution g is regarded as the output sequence. Define the nonlinear correspondence:

(11) $$g=f_{\mathrm{TFM}}\left(c\right)$$

Among them, *f*_TFM_ is a nonlinear function established by the Transformer model. The whole calculation process no longer depends on a linear hypothesis and fully takes advantage of the number of independent measurements brought by ER-ECT.

The calculation process of the self-attention mechanism of the Transformer model is:

(12) $$\mathrm{Attention}\left(Q,K,V\right)=\mathrm{softmax}\left(QK^{T}/\sqrt{d_{k}}\right)V$$

where *Q* = *W_q_x*, *K* = *W_k_x*, *V* = *W_v_x* are the query, key, and value vectors, respectively, *x* is the input embedding, *d_k_* is the dimension of the key vector, and $\sqrt{d_{k}}$ prevents the value from being too large.

Multi-head attention can be calculated in parallel by multiple attention to increase the extraction ability of the overall feature, which is equivalent to the division of each attention operation, to extract feature information from multiple low-latitude subspaces:

(13) $$\mathrm{MultiHead}\left(Q,K,V\right)=\mathrm{Concat}\left({\mathrm{head}}_{1},\ldots ,{\mathrm{head}}_{h}\right)W^{o}$$

where ${\mathrm{head}}_{h}=Attention\left(Q,K,V\right)$. Through the self-attention mechanism, 198 independent measurements in ER-ECT can be linked together to participate in mapping of the dielectric distribution *g*. By dynamically allocating the attention weight *a_ij_*, the complex relationship between these measurements can be identified, which is similar to the role of the sensitivity matrix. The nonlinear characteristics of the data can be retained without linear assumptions.

Because the Transformer model itself cannot sense the sequence order, and the spatial position of the electrode in the ECT measurement determines the electric field distribution of the area to be measured, the position coding is introduced:

(14) $$PE_{\left(pos,2k\right)}=\sin\left(pos/{10,000}^{2k/d}\right)$$

(15) $$PE_{\left(pos,2k+1\right)}=\cos\left(pos/{10,000}^{2k/d}\right)$$

where *pos* represents the serial number of independent measurements, *d* is the dimension of position coding, and the two equations represent odd and even positions, respectively.

In the field of natural language processing, the Transformer completes its learning process through massive data training, and its training complexity and model parameters are more complex than simple neural network algorithms. For the research of this paper, its training data depends on simulation data and self-collection. Insufficient sample size may lead to over-fitting in complex neural networks. Therefore, this paper uses Dropout to reduce the dependence on feature detection and reduce the risk of over-fitting. Dropout is the probability that the neurons in the model are set to zero. This probability is a hyperparameter called dropout rate, which can force the neural network to learn a more robust feature representation in the trainer, which helps to reduce the dependence on specific neurons and improve its generalization ability. The process is as follows:

(a) The random neurons were temporarily removed in the Dropout layer, but the neurons in the input and output layers remained unchanged.
(b) The adjusted network will input the data forward propagation to calculate the loss, and the loss result will be back-propagated. After processing a batch of training samples, the parameters of the neurons that have not been removed are updated.
(c) At the beginning of each iteration, the removed neurons are added to the network recovery state, and the unremoved neurons continue to be updated. Then, a part of the neurons is randomly selected to be removed again, and the iteration is repeated.

**Figure 2:**
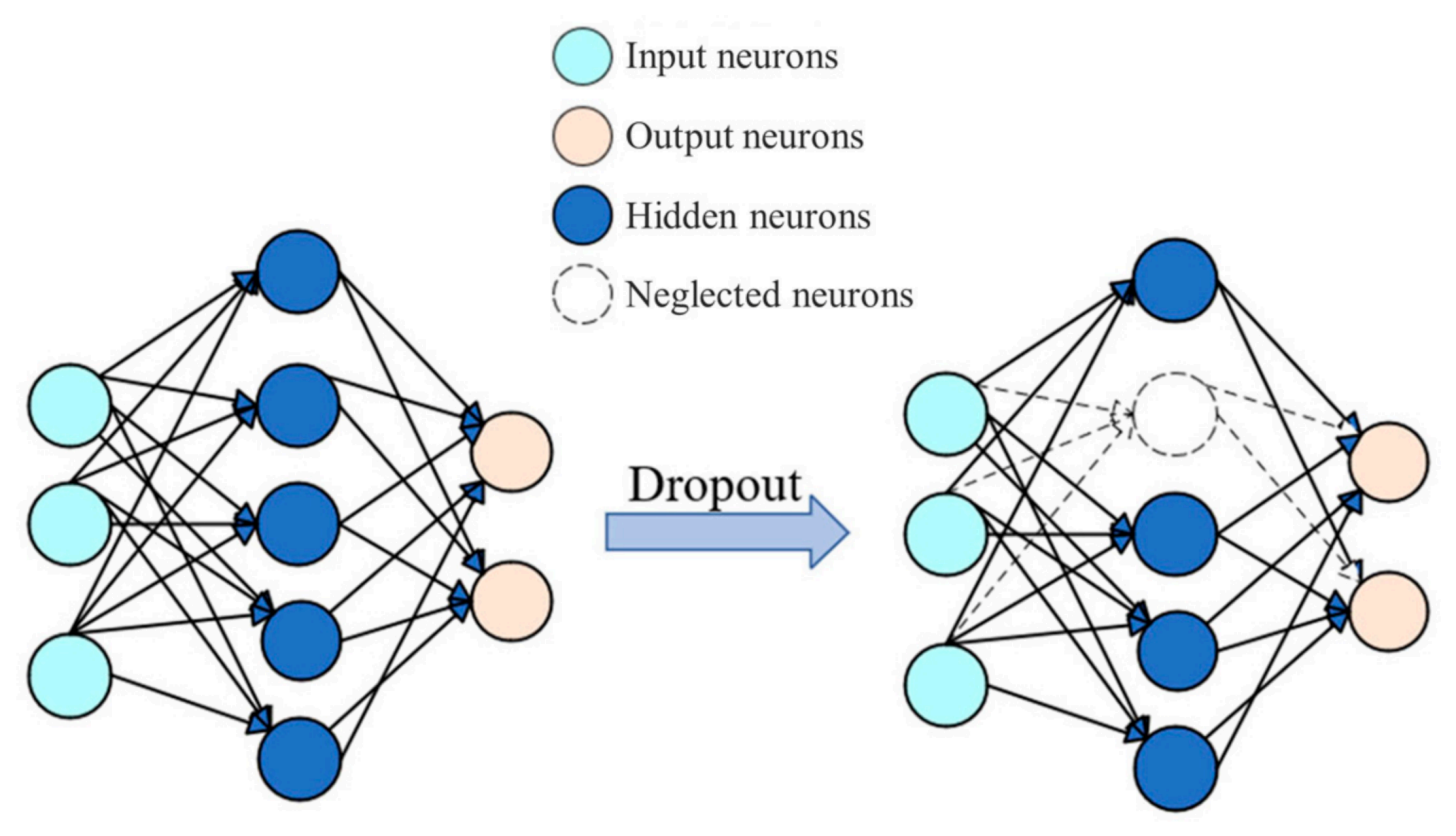
Dropout layer work diagram.

As shown in Figure 2, this Dropout method is a regularization method, so the loss function of ER-ECT-T can use mean square error. The larger the loss function, the less robust the model is. However, it is one of the most effective means of testing the model’s training effect.

The adaptive moment estimation updates the parameters of the ER-ECT-T model, an iterative algorithm with a fast convergence speed. Based on the gradient descent method, the first and second-moment estimates of the gradient are used to adjust each weight. The dependence on the initial learning rate is small, effectively reducing the time for hyperparameter adjustment.

**Figure 3:**
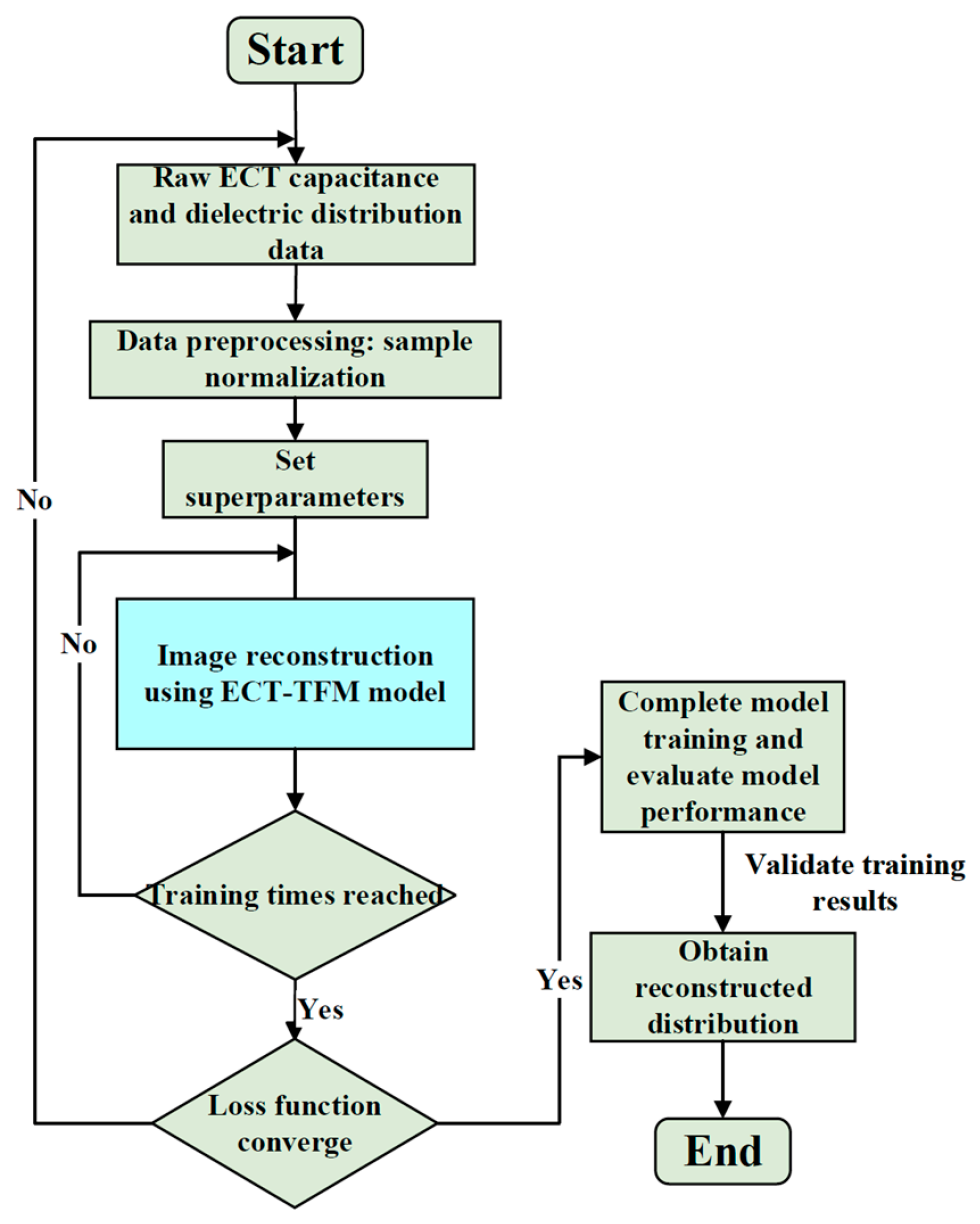
The flow chart of the ER-ECT-T algorithm.

The above method is used to regularize and optimize the ER-ECT-T model to make it suitable for the ECT measurement method. The learning process can be completed normally in the case of a small number of samples. Figure 3 shows the processing flow of the ER-ECT-T algorithm.

## 3. Results

### 3.1. ER-ECT Simulation Results

This paper designed four static phantoms to verify the imaging effect of ER-ECT and simulate various flow regimes in the imaging system: annular flow, core flow, eccentric flow, and stratified flow. The simulation measurement data are obtained by COMSOL Multiphysic 6.0 modeling. Linear back projection (LBP), Landweber iteration (LIA), and Tikhonov regularization (TRM) are used to reconstruct the image, and the reconstruction effect of traditional 12-electrode and ER-ECT is compared.

**Figure 4:**
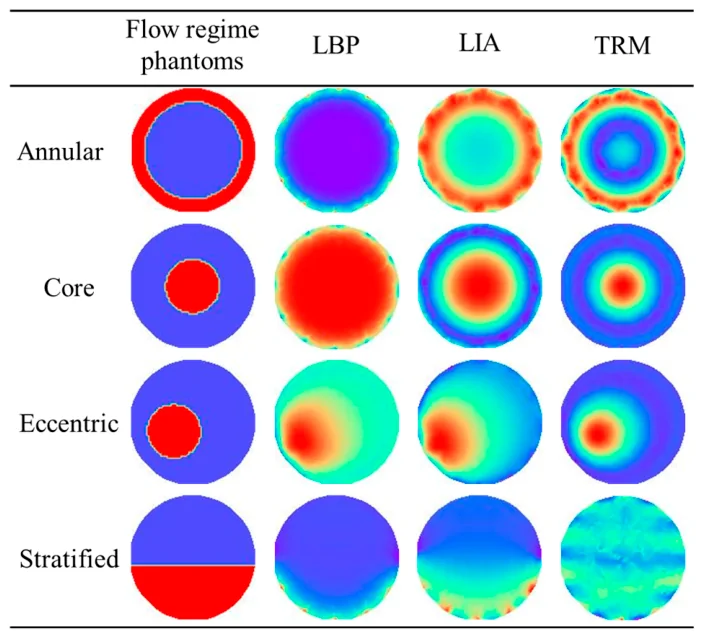
The image reconstruction results of traditional 12-electrode ECT.

Figure 4 shows that the LBP algorithm has the worst imaging effect, cannot characterize the dielectric constant distribution of the cross-section, and cannot distinguish the four flow regime phantoms. The LIA and TRM can roughly see the distribution of the medium, but there are serious artifacts. The edges show a wave-like distribution, and the edge part cannot be distinguished. There is a large measurement noise, and the actual distribution cannot be accurately characterized.

**Figure 5:**
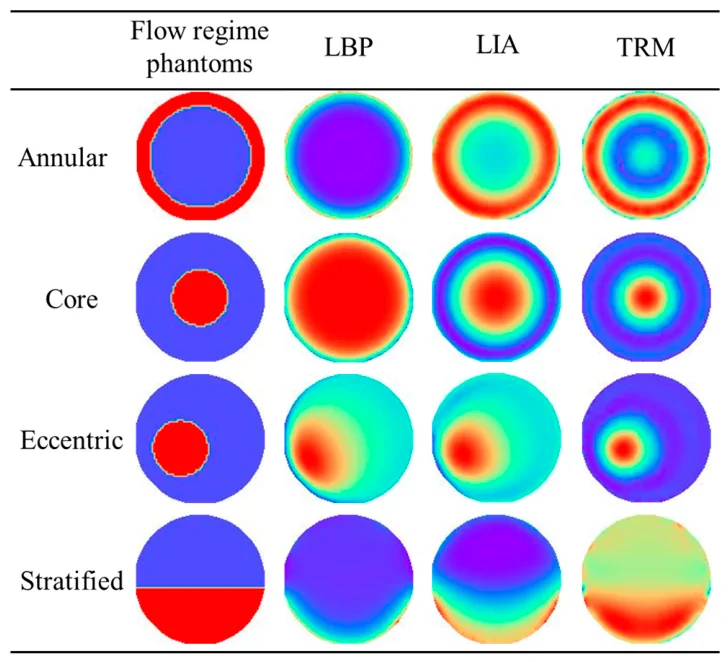
The image reconstruction results of ER-ECT.

Figure 5 shows the processing results of ER-ECT under the same image reconstruction algorithm. Compared with the traditional 12-electrode ECT, the imaging effect of ER-ECT in the edge part is significantly improved, and the wavy edge is almost invisible. It can more accurately characterize the cross section’s dielectric constant distribution, and the reconstructed image’s quality and accuracy are greatly improved. However, a specific gap exists between the image reconstruction effect and the flow regime phantom for stratified flow.

In order to evaluate the error between the reconstructed image and the flow regime phantom, the relative error is used to measure the quality of the reconstructed image:

(16) $$e_{img}\left(x,y\right)={\left|\left|x-y\right|\right|}_{2}/{\left|\left|y\right|\right|}_{2}$$

From the data in Table 1 and Table 2, the average error of ER-ECT is 11.27% lower than that of ECT for annular flow. For the core flow, it is reduced by 11.13%; for the eccentric flow, it is reduced by 15.08%. For stratified flow, it is reduced by 5.26%. The ER-ECT reconstructed image of the combined excitation measurement has a minor relative error with the flow regime phantom compared to the traditional 12-electrode ECT reconstructed image. The ER-ECT technology proposed in this paper improves the image reconstruction quality problem caused by the underdetermined problem.

To clarify the choice of subdivision number, the influence of different subdivision numbers on system performance is analyzed. Theoretically, a larger number of subdivided electrodes provides more independent measurements, which further alleviates the underdetermined problem of ECT and improves reconstruction performance.

Simulation experiments are carried out based on the average RE metric adopted in this work, and the results are presented in Table 3.

As the number of subdivided electrodes increases, the reconstruction performance is gradually improved. However, in practical hardware implementation, when the subdivision number reaches 4, each secondary electrode becomes excessively small. This introduces significant circuit interference to the existing ECT system and greatly increases the difficulty of electrode fabrication and circuit implementation, which exceeds the capacity of current experimental conditions. Therefore, three subdivided electrodes are employed for each electrode plate, achieving a favorable trade-off between reconstruction performance and hardware feasibility.

### 3.2. ER-ECT and ER-ECT-T Test

In this paper, a simulation model is adopted to construct the sample dataset of “capacitance–permittivity” under ideal conditions. In the simulation, the mixture of liquid and air is regarded as a two-phase flow, whose relative permittivity is set to 10 and 1, respectively, and 198 independent measurements of ER-ECT can be collected. The inner and outer radii of the pipeline are 25 mm and 28 mm, and the interior is subdivided into 3064 mesh elements. COMSOL is used to generate samples via the finite element method, including constructing the geometry of the capacitance sensor in the software and setting the boundary conditions according to (4). Then, combined excitation is applied following the ER-ECT principle described in Section 2.1 to calculate the capacitance values at the finite element mesh nodes. In this way, the capacitance values at the grid points required for image reconstruction can be obtained. Different flow regime phantoms can be obtained by setting different combinations of relative permittivity in the subdivided mesh.

The finite element simulation software is used to model the ER-ECT system, and four standard industrial flow regime phantoms are generated. For each flow regime phantom, 2000 “capacitance-dielectric distribution” samples were obtained, a total of 8000 samples. Among them, 400 samples were randomly selected from each flow regime phantom and combined as the network’s test set.

**Figure 6:**
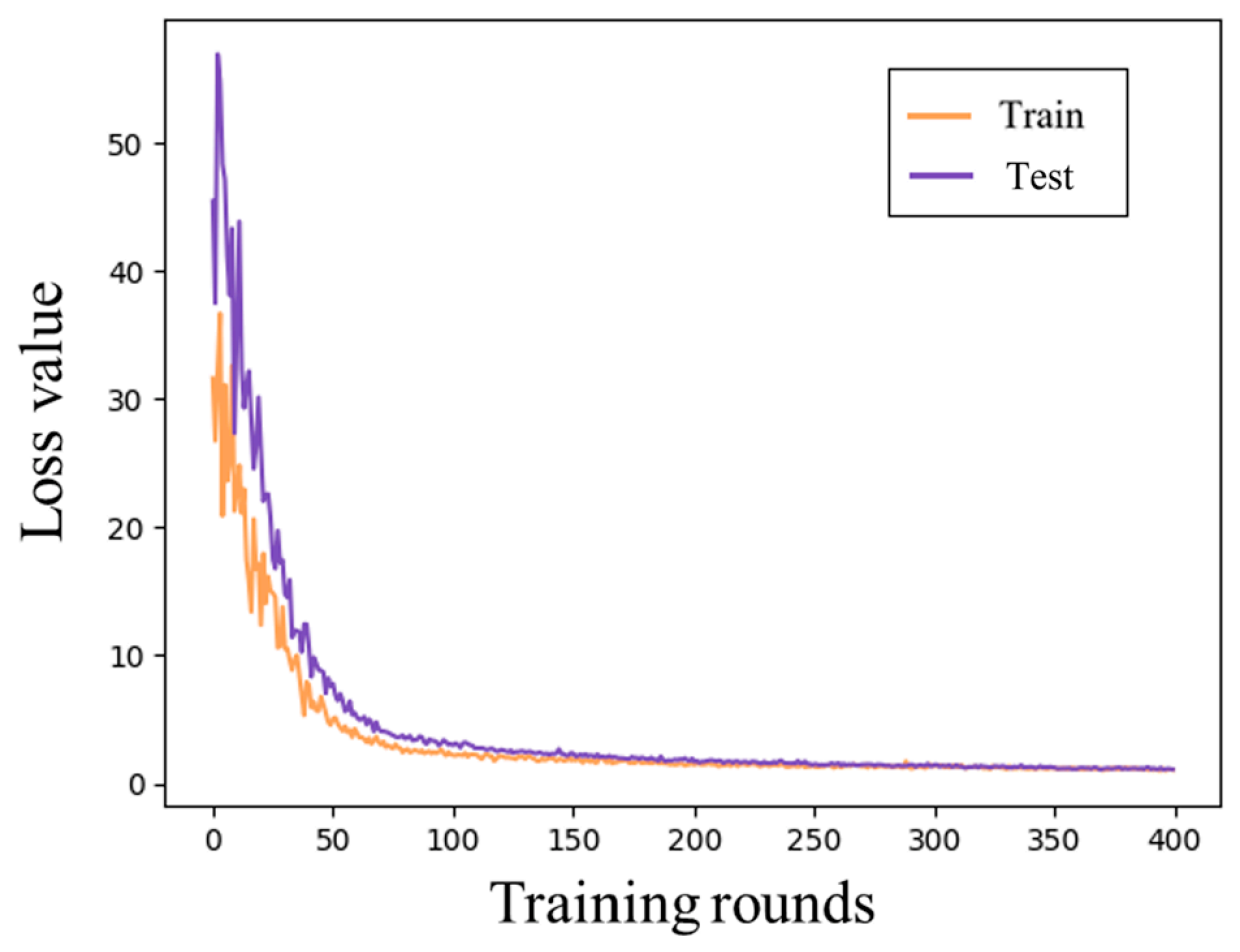
Loss curves during training versus epochs.

The model’s multi-head attention heads are set to 6, the stack encoder layers are 4, the dropout rate is 0.2, and the learning rate is 0.001. During the test, the capacitance vector is used as the input, the output layer is still the medium distribution vector, and the iteration rounds are set to 400 times. The obtained training loss value is shown in Figure 6.

To further verify the superiority of the proposed method, a stacked autoencoder (SAE) is introduced for ER-ECT image reconstruction [17]. As a typical deep learning model, SAE has been widely adopted in data compression and image generation applications in recent years. The proposed ER-ECR-T is compared with the SAE model, where both are representative methods in deep learning.

**Figure 7:**
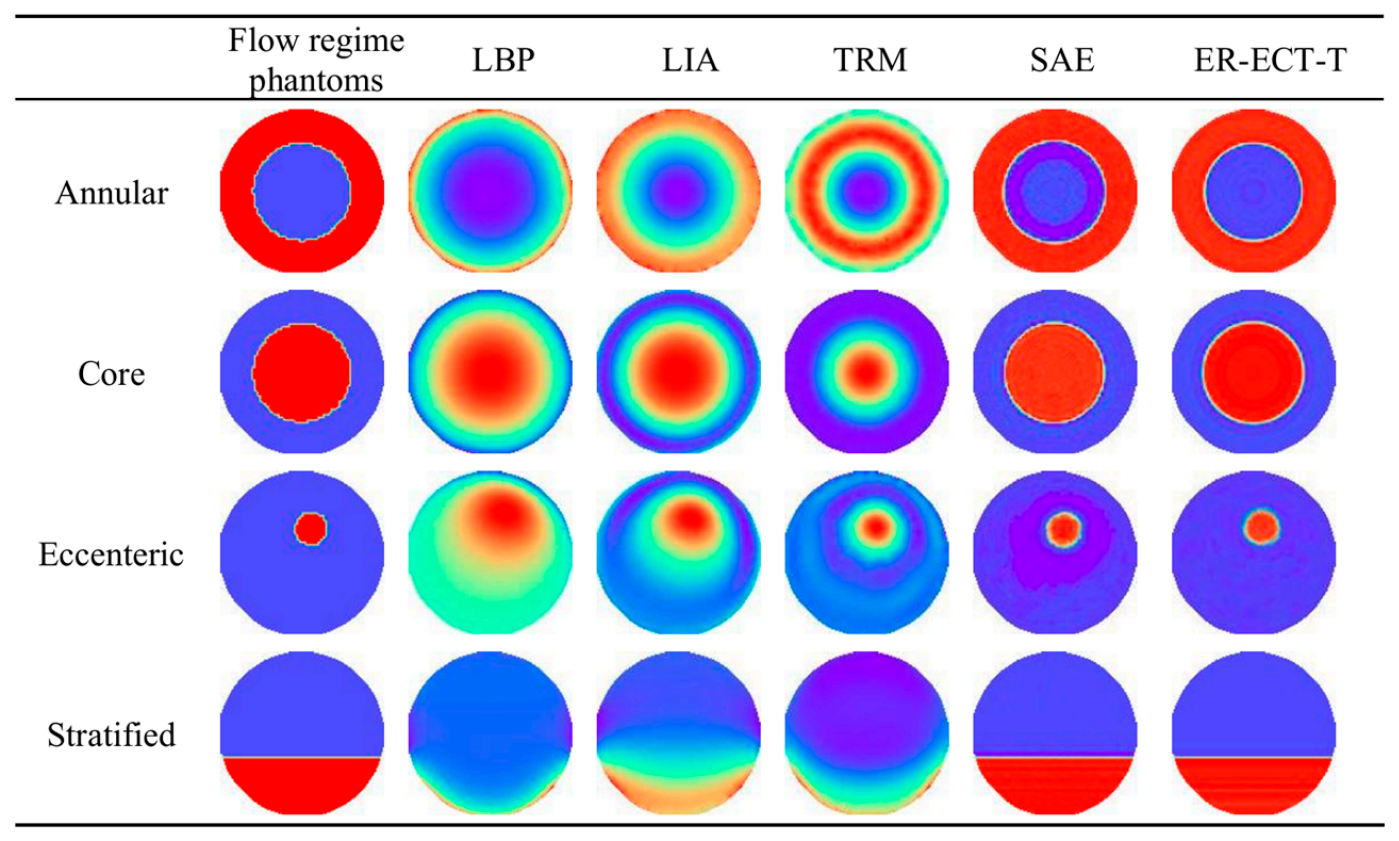
The image reconstruction results of different methods.

The reconstruction results of the two methods and traditional algorithms are illustrated in Figure 7, which demonstrates that the deep learning methods achieves more accurate and stable reconstruction performance than traditional algorithms. Meanwhile, ER-ECT-T still achieves the function of significantly reducing artifacts and presenting flow regime phantom characteristics and medium distribution more clearly. Like the NLP model, the Transformer model only learns 2000 samples in a small data set, and the reconstruction effect is still excellent.

This paper quantitatively describes the image reconstruction effect of ER-ECT-T using three indexes: correlation coefficient (CC), image relative error (RE), and structural similarity (SSIM).

The image correlation coefficient expresses the degree of linear correlation between image vectors. Using the standard Pearson correlation coefficient, its expression is:

(17) $$\mathrm{CC}\left(x,y\right)=\frac{\sum_{i=1}^{n}\left(x_{i}-\bar{x}\right)\left(y_{i}-\bar{y}\right)}{\sqrt{\sum_{i=1}^{n}{\left(x_{i}-\bar{x}\right)}^{2}\sum_{i=1}^{n}{\left(y_{i}-\bar{y}\right)}^{2}}}$$

The linear relationship is stronger when the correlation coefficient is close to 1. Structural similarity is used to measure the similarity of two pictures:

(18) $$\mathrm{SSIM}\left(x,y\right)=\frac{\left(2{\bar{u}}_{x}{\bar{u}}_{y}+c_{1}\right)\left(2\sigma_{xy}+c_{2}\right)}{\left({\bar{u}}_{x}^{2}+{\bar{u}}_{y}^{2}+c_{1}\right)\left(\sigma_{x}^{2}+\sigma_{y}^{2}+c_{2}\right)}$$

The value range of SSIM is [−1, 1], ${\bar{u}}_{x}$ and ${\bar{u}}_{y}$ represent the mean values of *x* and *y,* respectively, $\sigma_{xy}$ represents the covariance of *x* and *y*, $\sigma_{x}^{2}$ and $\sigma_{y}^{2}$ represent the variance of *x* and *y*, $c_{1}$ and $c_{2}$ represent the nonzero constants related to the pixel range.

Table 4, Table 5 and Table 6 show the results of ER-ECT-T compared with LBP, LIA, TRM, and SAE methods.

After comparing the five image reconstruction effects through Table 4, Table 5 and Table 6, it can be seen that traditional algorithms have a poor perception of the distribution of stratified flow among the four flow regime phantoms, reflecting the deep learning models’ advantages in image reconstruction of specific flow regime phantoms. In the comparison between the two deep learning models, the ER-ECT-T model incorporates additional boundary feature extraction modules, which delivers superior boundary representation performance and achieves higher reconstruction image quality than algorithms without attention mechanisms such as SAE.

In order to test the robustness of ER-ECT-T to noise, different degrees of white noise are added to the test data samples for simulation experiments, and the reconstructed image and relative error are obtained.

**Figure 8:**
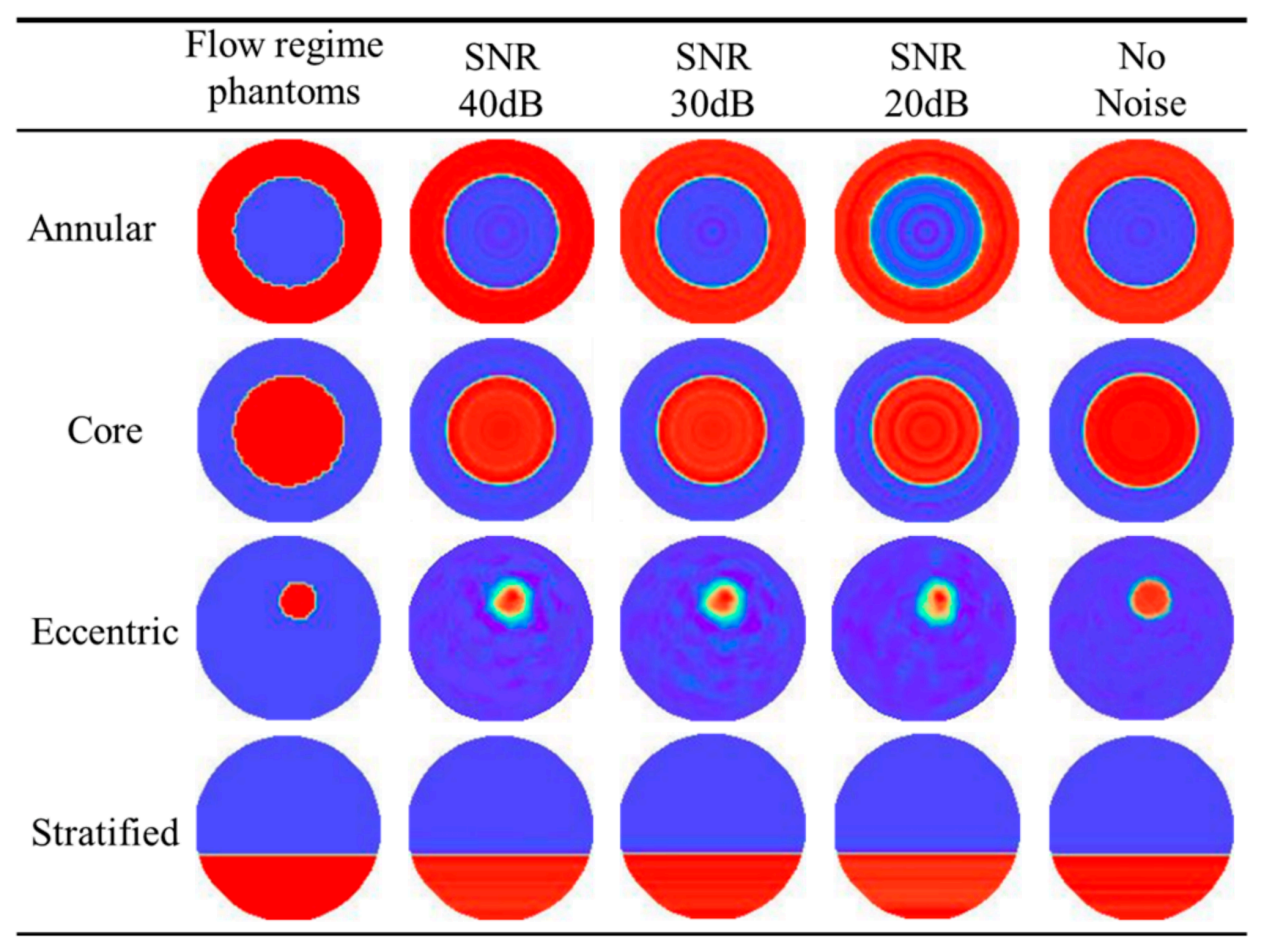
The influence of noise on ER-ECT-T.

According to Figure 8 and Table 7, after adding noise with different signal-to-noise ratios to the data set, the ER-ECT-T model can still well reflect the medium distribution inside the pipeline, and the boundary is still apparent. In addition to the slight increase in the reconstruction of eccentric flow, the artifacts of other flow regime phantom images are still very few. When the signal-to-noise ratio is above 20 dB, the correlation coefficient between the reconstructed image of ER-ECT-T and the target image is still above 0.90, especially for the layered flow, and the change of the correlation coefficient is at the level of 10-4. ER-ECT-T performs very well in the reconstruction of layered flow images.

To verify the model’s generalization ability, one of the four flow regime phantoms is completely removed from the training set in sequence. The model is trained solely on data from the remaining three phantoms, and the data corresponding to the removed flow pattern are then used for testing. The reconstruction quality under leave-one-class-out validation is summarized in Table 8.

The results demonstrate that the ER-ECT-T model maintains superior reconstruction performance on entirely unseen flow regimes. The average CC reaches 0.944, with an average RE of 0.132. Compared with the initial test method in this paper, the error increases by about 4%, which remains within a controllable range.

**Figure 9:**
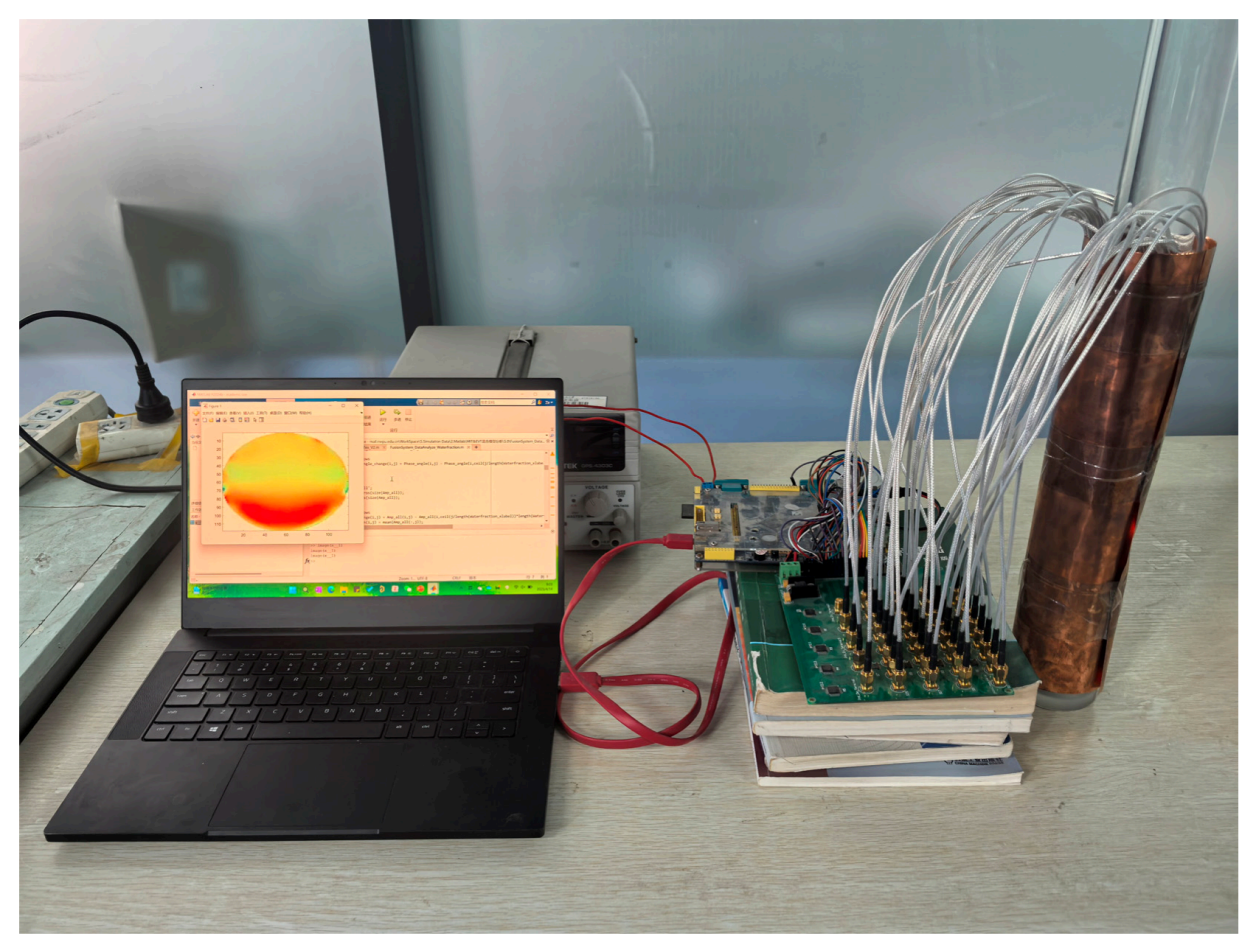
The physical picture of ER-ECT sensor.

This paper designs a static test to verify the performance of ER-ECT. Construct the core, eccentric, and stratified flow for the image reconstruction test. Figure 9 is the physical diagram of the ER-ECT system developed by the research team. The experimental materials are distilled water and air, and the dielectric constants are 81 and 1, respectively.

**Figure 10:**
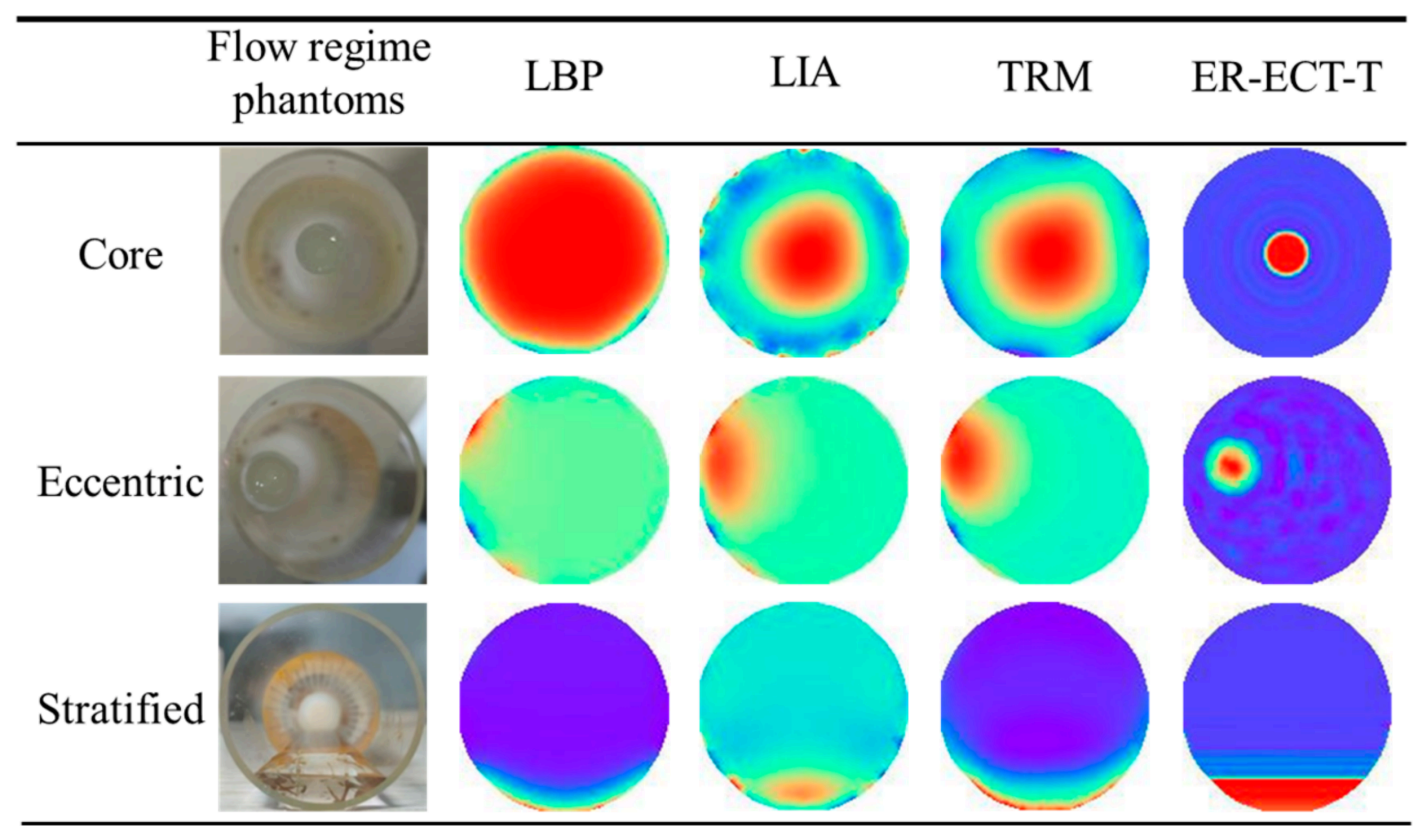
The image reconstruction results of ER-ECT-T in experimental environment.

The FPGA board connects with the ER-ECT sensor, collects sensor data, and transmits them to the PC side. Using the ER-ECT-T model obtained by training, various reconstruction algorithms calculate the measured capacitance input data, and the reconstruction effect of the ER-ECT-T model reconstruction algorithm and LBP, LIA, and TRM is compared. The results are shown in Figure 10.

Compared with LBP, LIA, and TRM algorithms, ER-ECT-T has fewer artifacts and clearer boundaries. In the case of eccentric flow, it can more clearly reflect the medium distribution of the cross-section of the two-phase flow system than the numerical method. There is no large area of adhesion between the artifacts and the pipe wall, and the performance of the layered flow is still significantly better than that of the numerical method.

The images reconstructed by different algorithms are quantitatively evaluated, and their CC and RE values are calculated, as shown in Table 9 and Table 10.

In the experimental environment, the correlation coefficient index of the reconstructed image using the ER-ECT-T method is much higher than that of the numerical method, and the relative error is also much smaller than that of the other three methods. For the presentation of laminar flow, the correlation coefficient can reach more than 0.98, which indicates that ER-ECT-T performs well in the reconstruction of laminar flow and is less affected by the error caused by actual working conditions.

## 4. Conclusions

This paper aims to address the underdetermined image reconstruction problem in ECT and propose an improved scheme based on ER-ECT. Combining the excitation plates and optimizing the measurement method increases the number of independent measurements to 198, and the imaging efficiency is maintained. In contrast, the number of plates is significantly increased, effectively alleviating the underdetermined problem of the traditional ECT system. Further, this paper uses linear back projection (LBP), linear iterative algorithm (LIA), and the Tikhonov regularization method (TRM) for image reconstruction of traditional 12-electrode ECT and ER-ECT, respectively, and takes relative error as the evaluation index. The comparative analysis of four typical flow regime phantoms of annular flow, core flow, eccentric flow, and layered flow shows that the average error of ER-ECT is 11.27%, 11.13%, 15.08%, and 5.26% lower than that of traditional ECT, respectively. Furthermore, the reconstruction performance of ER-ECT-T is compared with the deep learning model Stacked Autoencoder (SAE). Results show that ER-ECT-T possesses a stronger ability to capture sequential boundary features.

In this paper, the ECT image reconstruction problem is analogous to the machine translation problem and, combined with the latest developments in artificial intelligence, proposes an ER-ECT-T model based on the Transformer model. By optimizing the network parameter update method and appropriately reducing the number of parameters, the model can adapt to the ER-ECT data set with a small sample size. The experimental results show that the ER-ECT-T model has excellent performance in noise robustness tests and static experimental reconstruction, and the correlation coefficient with the test image can reach more than 0.89, which proves its effectiveness in the ECT image reconstruction task.

However, deep learning models usually require many data for practical training. Research teams do not share the data sets, insufficient training samples, the public ECT data sets are unavailable, and there is no common standard for measurement methods, which affects the model’s performance and ability to generalize. In addition, as a novel cross-disciplinary direction including hardware and algorithm improvement, this field currently has very limited related research. Most existing deep learning-based ECT works only focus on software algorithm optimization, with few efforts on hardware upgrading, which means we have very few reference methods in this new direction. Therefore, the self-measured data set in this paper can be further optimized and extended by data augmentation and data augmentation techniques to generate more samples quickly. And we will further adapt the state-of-the-art methods to the ER-ECT system and present a more comprehensive comparison in our follow-up work.

As an imaging technology based on physical principles, ECT’s core is the physical relationship between the dielectric constant distribution and the measured data. Therefore, an important direction for subsequent research is to incorporate physical constraints into deep learning models to ensure the physical rationality of the reconstruction results and improve the reliability and applicability of the model.

## Figures and Tables

**Table 1:** Relative error of traditional 12-electrode ECT.

Flow Regime Phantoms	RE		
	LBP	LIA	TRM
Annular	0.895	0.601	0.572
Core	0.842	0.752	0.554
Eccentric	0.934	0.709	0.598
Stratified	0.955	0.557	0.673

**Table 2:** Relative error of ER-ECT.

Flow Regime Phantoms	RE		
	LBP	LIA	TRM
Annular	0.827	0.524	0.484
Core	0.804	0.570	0.535
Eccentric	0.903	0.510	0.491
Stratified	0.890	0.541	0.639

**Table 3:** The correlation coefficient of ER-ECT.

The Number of Subdivided Electrodes	Independent Measurements	Average RE
1	66	0.720
2	132	0.687
3	198	0.643

**Table 4:** The correlation coefficient of different methods.

Flow Regime Phantoms	CC				
	LBP	LIA	TRM	SAE	ER-ERC-T
Annular	0.5866	0.6461	0.6721	0.9712	0.9934
Core	0.7297	0.7444	0.6981	0.9933	0.9907
Eccentric	0.6333	0.7843	0.8497	0.9746	0.9846
Stratified	0.6264	0.7257	0.6517	0.9934	0.9997

**Table 5:** The relative error of different methods.

Flow Regime Phantoms	RE				
	LBP	LIA	TRM	SAE	ER-ERC-T
Annular	0.8397	0.5231	0.4821	0.0980	0.0631
Core	0.7931	0.5646	0.5380	0.0875	0.0981
Eccentric	0.8854	0.5133	0.4976	0.1026	0.1004
Stratified	0.8752	0.5312	0.6294	0.0342	0.0413

**Table 6:** The structural similarity of different methods.

Flow Regime Phantoms	SSIM				
	LBP	LIA	TRM	SAE	ER-ERC-T
Annular	0.3506	0.4564	0.4162	0.6306	0.7407
Core	0.4870	0.5520	0.5735	0.7941	0.8031
Eccentric	0.4985	0.5896	0.7160	0.8250	0.9178
Stratified	0.6898	0.7372	0.7253	0.9171	0.9246

**Table 7:** The correlation coefficient of ER-ECT-T under different noises.

Flow Regime Phantoms	CC			
	SNR 40 dB	SNR 30 dB	SNR 20 dB	No Noise
Annular	0.9930	0.9714	0.9452	0.9934
Core	0.9886	0.9889	0.9851	0.9907
Eccentric	0.9542	0.9392	0.9008	0.9846
Stratified	0.9995	0.9994	0.9994	0.9997

**Table 8:** The quality of ER-ECT-T reconstruction under leave-one-class-out validation.

Removed Flow Regimes	Average CC	Average RE	Average SSIM
Annular	0.952	0.125	0.712
Core	0.943	0.137	0.755
Eccentric	0.925	0.150	0.871
Stratified	0.954	0.118	0.880

**Table 9:** The correlation coefficient of ER-ECT-T.

Flow Regime Phantoms	CC			
	LBP	LIA	TRM	ER-ERC-T
Core	0.4901	0.5748	0.6012	0.9451
Eccentric	0.2852	0.4121	0.5922	0.8925
Stratified	0.3414	0.5110	0.5517	0.9803

**Table 10:** The relative error of ER-ECT-T.

Flow Regime Phantoms	RE			
	LBP	LIA	TRM	ER-ERC-T
Core	0.8524	0.6131	0.5439	0.1491
Eccentric	0.8631	0.6551	0.5814	0.1621
Stratified	0.8994	0.6247	0.5826	0.1005

## Data Availability

The original contributions presented in this study are included in the article. Further inquiries can be directed to the corresponding author.
